# Supplementary material for: DMRforPairs: identifying Differentially Methylated Regions between unique samples using array based methylation profiles
Source: BMC Bioinformatics. 2014 May 15;15:141. doi: 10.1186/1471-2105-15-141 (PMC4046028; doi:10.1186/1471-2105-15-141)
Supplement: Additional file 2 — DMRforPairs output for the comparison of A431-MCF7 and NA17018-NA17105. Please start from the HTML files in each folder. Available via the BMC Bioinformatics website. [file 1471-2105-15-141-S2.zip › 1394847754114233_MOESM2_ESM/A431_MCF7/figures/13594.pdf]

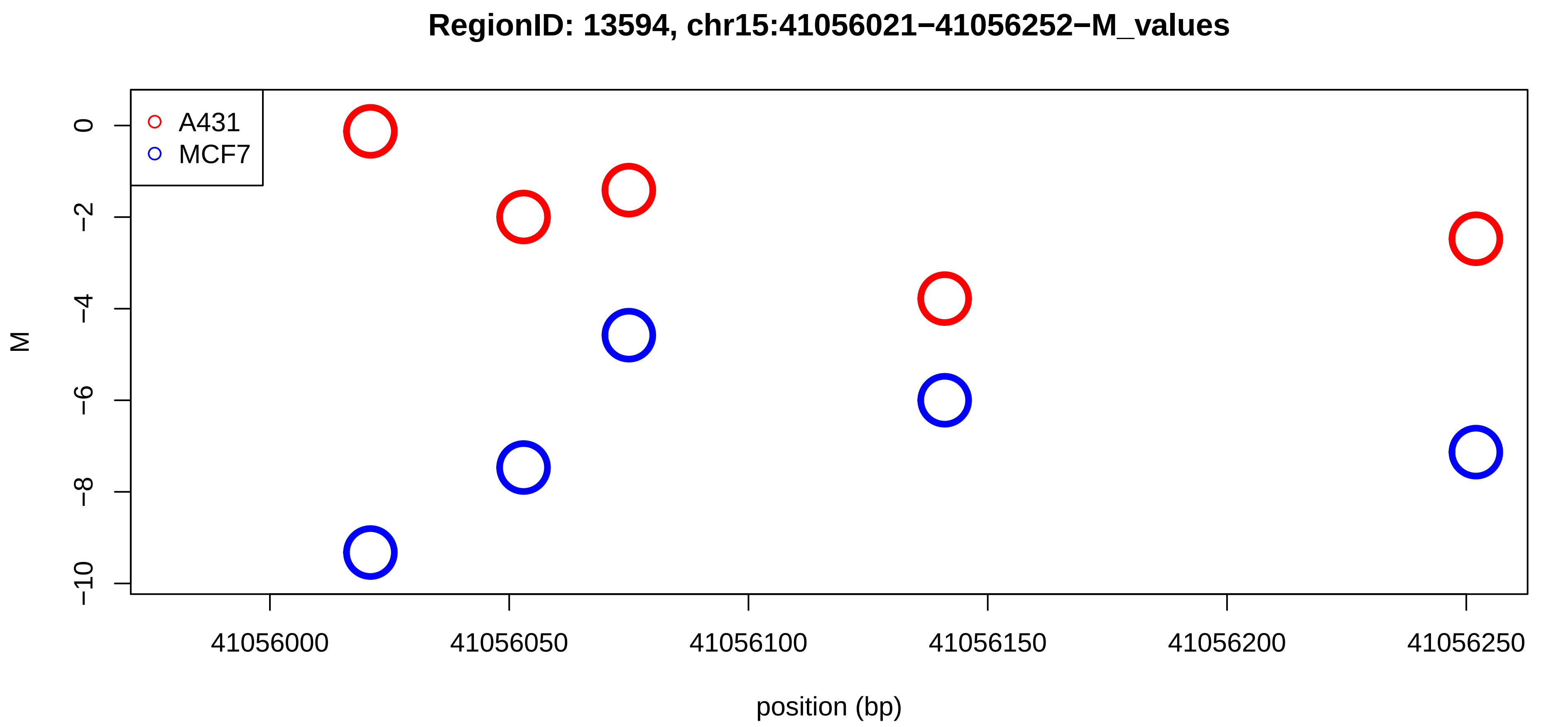
RegionID: 13594, chr15:41056021−41056252−M_values
A431
MCF7
M
0
−2
−4
−6
−8
−10
41056000
41056050
41056100
41056150
41056200
41056250
position (bp)

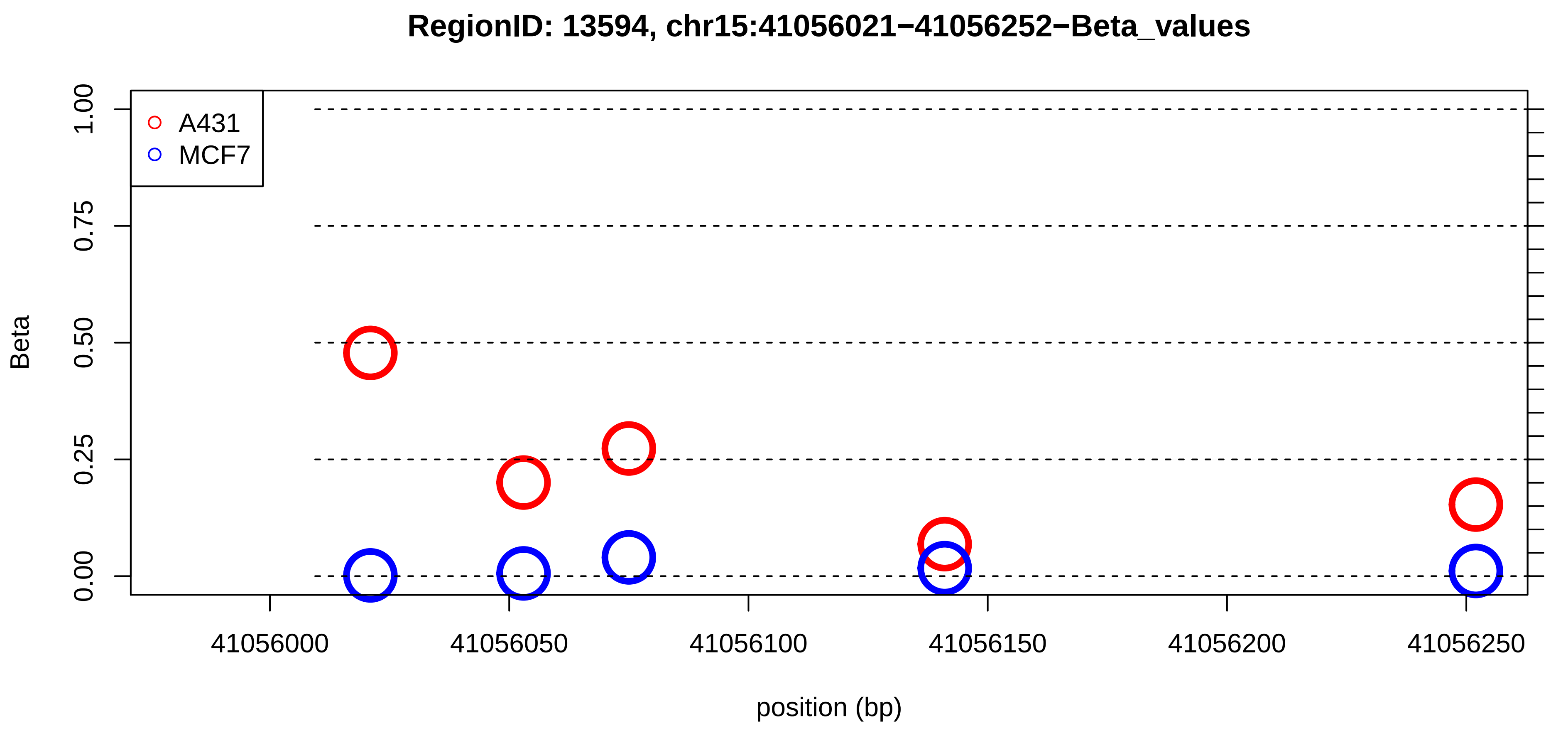
RegionID: 13594, chr15:41056021−41056252−Beta_values
A431
MCF7
Beta
1.00
0.75
0.50
0.25
0.00
41056000
41056050
41056100
41056150
41056200
41056250
position (bp)

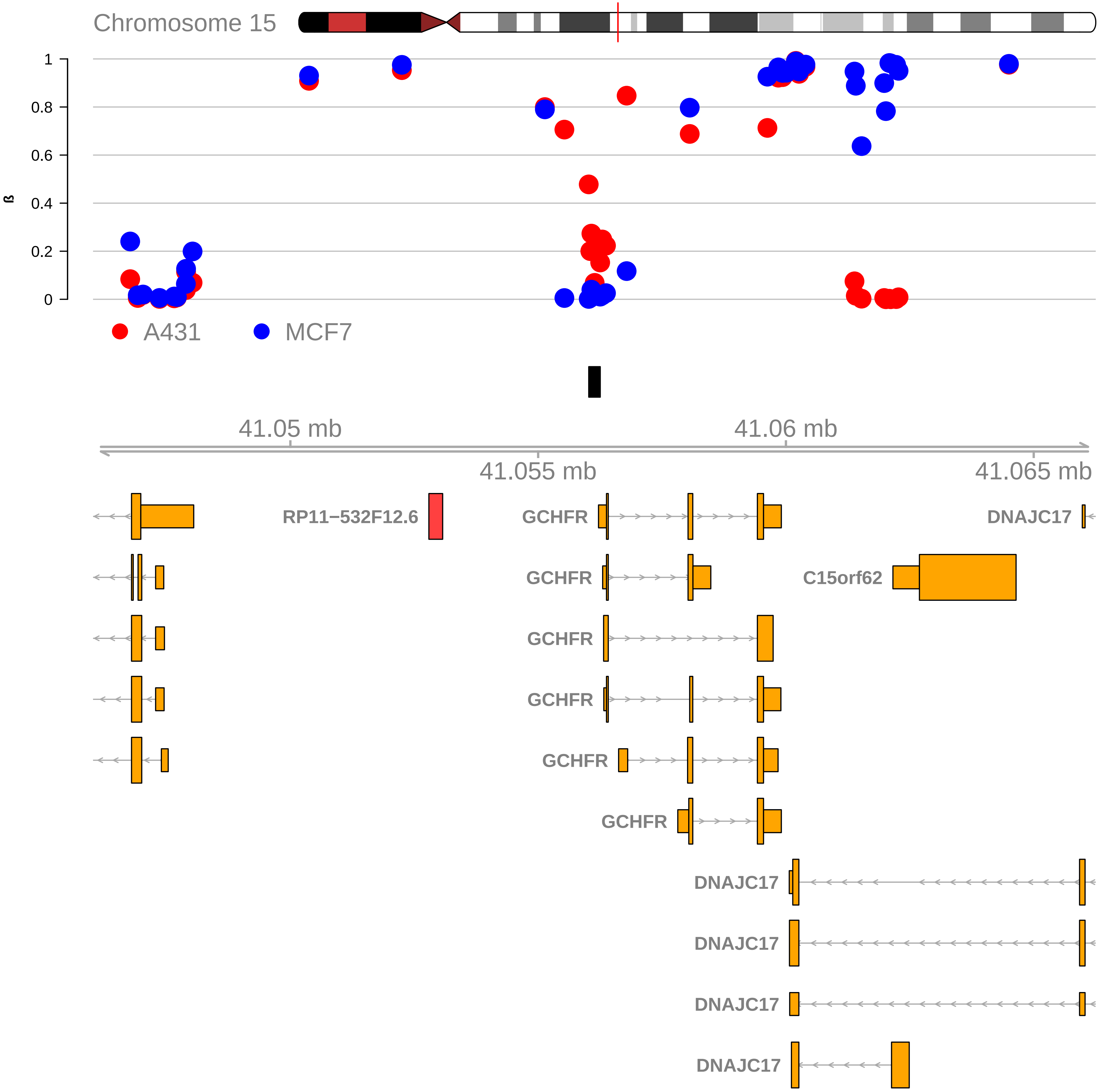

Chromosome 15
1
0.8
0.6
ß
0.4
0.2
0
A431
MCF7
41.05 mb
41.06 mb
41.055 mb
41.065 mb
RP11−532F12.6
GCHFR
DNAJC17
GCHFR
C15orf62
GCHFR
GCHFR
GCHFR
GCHFR
DNAJC17
DNAJC17
DNAJC17
DNAJC17
